# Making birth defects ‘preventable’: Pre-conceptional vitamin supplements and the politics of risk reduction^☆^

**DOI:** 10.1016/j.shpsc.2013.10.009

**Published:** 2014-09

**Authors:** Salim Al-Gailani

**Affiliations:** Department of History and Philosophy of Science, University of Cambridge, Free School Lane, Cambridge CB2 3RH, UK

**Keywords:** Folic acid, Vitamin supplements, Pre-conception, Public health, Clinical trials, Risk

## Abstract

Since the mid-1990s, governments and health organizations around the world have adopted policies designed to increase women’s intake of the B-vitamin ‘folic acid’ before and during the first weeks of pregnancy. Building on initial clinical research in the United Kingdom, folic acid supplementation has been shown to lower the incidence of neural tube defects (NTDs). Recent debate has focused principally on the need for mandatory fortification of grain products with this vitamin. This article takes a longer view, tracing the transformation of folic acid from a routine prenatal supplement to reduce the risk of anaemia to a routine ‘pre-conceptional’ supplement to ‘prevent’ birth defects. Understood in the 1950s in relation to social problems of poverty and malnutrition, NTDs were by the end of the century more likely to be attributed to individual failings. This transition was closely associated with a second. Folic acid supplements were initially prescribed to ‘high-risk’ women who had previously borne a child with a NTD. By the mid-1990s, they were recommended for all women of childbearing age. The acceptance of folic acid as a ‘risk-reducing drug’ both relied upon and helped to advance the development of preventive and clinical practices concerned with women’s health before pregnancy.

## Highlights

• Shows how folic acid supplements became part of the experience of pregnancy.
• These supplements became a technology for reducing risk in pregnancy in the 1980s.
• This identity as a ‘risk-reducing’ drug relied on the agency of non-experts.

## 1 Introduction: folic acid and risk reduction

Since the mid-1990s, governments and health organizations around the world have adopted policies designed to increase women’s dietary intake of ‘folic acid’ before and during pregnancy in order to reduce the risk of having a baby with a defect of the brain and/or spinal cord. Folic acid is the synthetic form of folate, a B-group vitamin naturally present in many foodstuffs, particularly leafy green vegetables, and presumed essential for cell growth and development. Neural tube defects (NTDs) such as spina bifida are among the most common congenital malformations and a significant cause of neonatal mortality worldwide (Blencowe, Cousens, Modell, & Lawn, 2010). The proposed link between dietary folate deficiency and birth defects has convinced several countries to fortify grain products with folic acid (Semba, 2006, p. 176). Others have relied on health education strategies to persuade women to take a daily supplement of folic acid in order to ‘help [their] baby develop a healthy brain and body’.^1^ At least for those women for whom pregnancy is planned, the decision to replace the daily ritual of swallowing a contraceptive pill with a vitamin supplement has come to signify the transition to ‘hoped-for motherhood’ and a more highly disciplined regime of consumption (Landsman, 2004, pp. 105–106; Taylor, 2008, p. 127). This article offers the first history of how taking a folic acid supplement became both a common part of the experience of pregnancy and a drug that promises to reduce individual risk.

Policies to encourage folic acid consumption are characteristic of the emphasis on risk prevention that came to dominate the management of health and disease in developed countries after 1945 (Berridge, 1999; Brandt, 1997; Rothstein, 2003). They are also a symptom of a broader trend in which the risk to the unborn fetus has displaced the risk of maternal mortality as the main focus of the medical interventions in pregnancy and childbirth (Lupton, 2012). Increased emphasis on the management of risk in childbirth since World War Two has justified the expansion of obstetric surveillance ever earlier into pregnancy (Arney, 1982, p. 143; Weir, 2006). However, risk prevention strategies have often developed in piecemeal fashion and cannot be attributed solely to professional aspirations to extend the scope of obstetric control. Studying the long-term trajectory of folic acid helps to recover the complex medical, social and political contingencies that helped to drive this shift.

Historians have long recognized that ‘risk-reducing’ interventions have not only enhanced the medical scrutiny of subjectively healthy populations, but also constituted novel categories of patients and disease states. Commercial promotion, professional enthusiasm and consumer demand for preventive medical interventions in chronic conditions such as cancer and diabetes have blurred the boundary between statistical risk and experienced illness, creating both symptomless ‘pre-patients’ and treatments for ‘proto-diseases’ (Aronowitz, 2009; Greene, 2007; Löwy, 2010; Rosenberg, 2007, p. 27; Valier, 2012). In a similar fashion, this article suggests, the acceptance of folic acid as a ‘risk-reducing drug’ both relied upon and helped to advance the development of a novel set of preventive and clinical practices concerned with women’s health before pregnancy. Originating around 1980, strategies to promote both ‘pre-conception’ or ‘pre-pregnancy’ care and ‘pre-conceptional’ vitamin supplementation evolved in close relation and were mutually reinforcing.

What kind of intervention is pre-conceptional vitamin supplementation? A complete answer would require an international and cross-disciplinary history over at least the last half-century. Research on folic acid was—and remains—global and multi-sited; individual clinical trials could span several countries, as we shall see. Yet even as the postwar globalization and homogenization of biomedical knowledge helped constitute folic acid as a risk-reducing drug, research into this vitamin and strategies to promote its consumption were also local initiatives, producing local responses. Expert disagreement over mandatory folic acid fortification has generated considerable international policy controversy over such issues as individual versus social responsibility, medical surveillance, the effectiveness of health education, and the risk and benefits of population-based interventions with unknown long-term consequences. This article puts these global debates aside for now in order to recover the ‘natural history’ of folate deficiency in pregnancy as a health risk.^2^ Instead, it focuses on the United Kingdom where much of the important research was done.

A British paediatrician, Richard Smithells, is credited with suggesting the ‘causal’ link between folate deficiency and NTDs, which a multi-centre clinical trial conducted by the UK Medical Research Council (MRC) is typically claimed to have confirmed. First tracing the history of folic acid in Britain, I propose that two factors significantly shaped this research and its local reception: one, the longstanding and widespread acceptance of vitamins, including folic acid, as beneficial to pregnant women; two, the rise to political prominence of spina bifida, the most common NTD, from the late-1960s. The second half of the article analyses the controversy around the MRC clinical trial and its immediate aftermath. This focus illuminates the multiple constituencies involved in the co-construction of folic acid as a risk-reducing drug and pre-conception care. I suggest that the controversy mobilized a heterogeneous set of actors—research scientists, clinicians, food and drugs companies, activists and journalists—with different interpretations of risk and uncertainty to claim a stake in pre-conceptional vitamin supplements as a means of preventing NTDs.

## 2 From Marmite to folic acid: vitamin supplements in pregnancy

Dietary prescriptions had long been a staple of informal health guidance to expectant and nursing mothers before rising concern about infant mortality around 1900 consolidated the status of nutritional advice in voluntary and statutory systems of maternal and child welfare (Cody, 2011; Dwork, 1987; Oakley, 1984). In the following decades, pregnant women were identified as important beneficiaries of the new knowledge of vitamins, deployed by not only medical practitioners and public health workers, but also charitable, educational and campaigning organizations. Manufacturers of ‘nutrient-rich’ proprietary foods such as Marmite, made from yeast extract, Stork margarine and the stabilized wheatgerm Bemax stressed that ‘vitamin need’ was ‘especially important in the case of the expectant mother, as any Maternity or Child Welfare centre will confirm’. The marketing of vitamin supplements as ‘indispensible’ for pregnant women, ‘for their own sake *and* that of the child’, extended a long tradition of emphasizing family nutrition as mothers’ responsibility and duty (Apple, 1995; 1996, p. 182).^3^

Many interwar health workers were convinced of the value of Marmite and other vitamin-rich preparations in reducing the risk of death in childbirth. By distributing Marmite and other supplements to working-class expectant mothers in England and Wales, the voluntary National Birthday Trust Fund and the People’s League of Health could combine relief with research into the impact of prenatal nutrition on maternal mortality (People’s League of Health, 1946; Williams, 1997). This conviction informed official wartime nutrition policies. The supply of free or cheap vitamin A and D tablets, milk, orange juice and cod liver oil to expectant mothers and children through the wartime vitamin welfare scheme, with their perceived role in the decline of maternal mortality, consolidated the importance of dietary supplements in pregnancy. At the war’s end, the Ministry of Food could declare that the national provision of milk and vitamin supplements to these ‘priority groups has probably done more than any other single factor to promote the health of expectant mothers and young children’ (quoted in Oakley, 1984, pp. 124–125; Sultan, 2010; Zweiniger-Bargielowska, 2000, pp. 130–134).

Like for other vitamins and vitamin products, the relations between medical researchers, philanthropists, industry and pregnant women in the 1930s helped constitute the therapeutic identity of folic acid (Kamminga, 1998). Working in India in the 1930s, the British pathologist Lucy Wills described a severe and often fatal anaemia associated with pregnancy that was especially prevalent among undernourished urban textile workers. The symptoms of ‘tropical macrocytic anaemia’ closely resembled those of pernicious anaemia, established by 1934 as a disease curable by liver extract (Wailoo, 1999, pp. 99–133). In research funded by the Lady Tata Memorial trust, founded by the Indian businessman Dorabji Tata in 1932, Wills and her collaborators studied the effects of dietary manipulation in albino rats, rhesus monkeys and hospital patients in Bombay and later in London. She observed that macrocytic anaemia could be treated effectively with both crude liver and yeast extract supplied by the Marmite Food Extract Company. This finding both differentiated the disease from pernicious anaemia and demonstrated the presence of a previously unknown ‘growth factor’ necessary for the formation of blood cells (Wills, 1933; Wills & Evans, 1938). The so-called ‘Wills factor’ of yeast extract and crude liver was later identified as a family of compounds that biochemists in Texas first isolated in 1941 by concentrating four tons of the spinach that gave folic acid its name (folium = leaf [Lat.]) (Mitchell, Snell, & Williams, 1941). Synthesized by chemists at the American pharmaceutical company Lederle in 1945, folic (also pteroylglutamic) acid quickly became a tool of clinical medicine and research (Hoffbrand & Weir, 2001).

Lederle’s ‘new yellow vitamin’ that ‘builds red blood cells’ and ‘helps anemic expectant mothers’ was one of many new technologies taken by haematologists to embody the promise of biochemical blood manipulation and analysis in the postwar era (Lederle, 1950).^4^ As Keith Wailoo has argued for liver extract in pernicious anaemia, the identities of folic acid and macrocytic (and later ‘megaloblastic’) anaemia, conditions particularly prevalent among pregnant women, were mutually constitutive. Understanding of these diseases depended upon not only the collaboration of clinicians and academic researchers, but also ‘the manufacturing, packaging, advertising, and promotion of the cure’ (Wailoo, 1999, p. 132). Postwar haematologists investigated the role of folate activity in the anaemias, especially in conjunction with vitamin B12, isolated from liver in the late-1940s. By around 1960, researchers had developed several techniques for diagnosing folate deficiency, including biochemical tests and animal and microbiological assays of blood serum, each of varying degrees of sensitivity and specificity.

One such method involved testing urine for abnormally high levels of ‘formiminoglutamic acid’ (FIGlu), an intermediate in the metabolic breakdown of the amino acid histidine, for which folate was understood to be integral. So by giving anaemic patients a large oral dose of histidine, increased FIGlu excretion offered a biochemical index for the diagnosis of clinical folate deficiency (Luhby, Cooperman, & Teller, 1959). Although the test was complex and proved difficult to interpret, high-voltage electrophoresis ‘greatly simplified’ the FIGlu assay (Hibbard, 1964; Metz, 1963, p. 526). Like other methods of blood analysis in this period, diagnostic tests for folate deficiency migrated beyond the expert province of haematology into other clinical disciplines (Wailoo, 1999, pp. 154–157, 162–187). Biochemical tools offered obstetricians and paediatricians, in particular, new ways of thinking about pregnancy.

By the mid-1960s, extensive clinical studies of the megaloblastic anaemias of pregnancy using such techniques brought into view a previously unknown problem: most pregnant women had mild folate deficiency whilst showing no signs of clinical disorder. Because of the now acknowledged role of folic acid in metabolism and cell growth, haematologists and obstetricians surmised that the developing fetus raised the physiological demand for the vitamin (Chanarin, Rothman, & Berry, 1965). Even otherwise healthy pregnant women needed supplementary folate to restore their reserves of the vitamin to ‘normal’ levels. In the postwar period, megaloblastic anaemias were common in ‘peoples with low standards of living in tropical and sub-tropical areas’, but rare in ‘developed’ countries like the UK. Metabolic studies of folate activity lent weight to the view that pregnant women were, in haematological terms, akin to the poorly nourished populations in the ‘underdeveloped’ world most vulnerable to this condition (Anon, 1964b, 1968b).

The fact that sub-clinical folate deficiency put pregnant women at ‘unnecessary risk’ of overt megaloblastic anaemia, moreover, justified the ‘ritual’ prescription of folic acid and iron supplements to expectant mothers at British antenatal clinics by the late 1960s (Anon, 1966; Today’s Drugs, 1967). Yet even as prenatal folic acid supplementation became ever more routine, some medical practitioners challenged the efficacy of a ‘technological fix’ to a condition that manifested clinically in only relatively few pregnant women. One clinician protested that this ‘blanket prescribing policy’ was not only expensive and ineffective (since many women did not take the supplements), but diverted attention from ‘the real problem’: improving nutritional education and influencing national policies so that the population as a whole was ‘more likely to eat a nutritionally sound diet’ (Smail, 1981).

Research into megaloblastic anaemia and pregnancy nevertheless stimulated medical interest in the wider role of folate metabolism in reproduction. Obstetricians looked beyond maternal anaemia to focus on the clinical manifestations of folate deficiency in the fetus and newborn, including abortion, prematurity and congenital malformations (Hibbard & Hibbard, 1968). By studying ‘tropical’ populations with diets known to be low in uncooked green vegetables, clinical researchers could increasingly claim folate deficiency as a cause of ‘reproductive wastage’ (Baumslag, Edelstein, & Metz, 1970). Such observations built on awareness that the folate antagonist aminopterin, a drug used to treat childhood leukaemia, was also an abortifacient (Anon, 1977; Thiersch, 1952), as well as a body of experimental research linking developmental abnormalities with vitamin-deficient diets. By starving pregnant laboratory animals of B-group vitamins riboflavin, pantothenic acid and folic acid, several teratologists had by around 1950 successfully produced ‘deformities … encountered as congenital malformations in man’ (Woollam & Millen, 1956, p. 1264).

Promoted since the 1930s as ideal nourishment for pregnant women, B-vitamin-rich yeast extract was in the 1960s claimed as beneficial for the developing fetus as well. The manufacturers of Marmite targeted medical practitioners in the developing world by advertising in the *Journal of Tropical Pediatrics*: babies, they claimed, needed ‘Marmite before birth’.^5^ While medical interest was by then directed at one of yeast extract’s molecular components, chemically synthesized folic acid, the rebranding of Marmite is a reminder of the multiple connections between the food industry, clinicians, academic scientists and consumers that have given vitamins and vitamin preparations in general such importance to medical and lay perceptions of health and disease since the early twentieth century. The postwar therapeutic identity of folic acid depended, in particular, on the longstanding emphasis on expectant mothers as especially appropriate consumers of vitamin supplements. Through new biochemical technologies, folic acid in turn forced obstetricians to revise their understanding of metabolism in pregnancy. This conjunction would, in the early 1980s, transform both folic acid and pregnancy once again.

## 3 Monitoring, treating and preventing NTDs after thalidomide

Revelations in the early 1960s that the anti-nausea drug thalidomide had caused birth deformities on a huge scale, prompted widespread discussion about the permissibility of abortion across Western Europe and North America. Although the movement for abortion law reform can be traced at least as far back as the 1930s, recent historical writing has stressed that the thalidomide disaster, together with publicity given to fetal abnormalities caused by rubella during global epidemics of 1962–1964, provided a major impetus to the liberalization of abortion (Parker, 2012; Reagan, 2010). The concurrence of abortion legalization and the development as diagnostic tools of chromosome analysis, amniocentesis, screening for serum markers and obstetric ultrasound facilitated the dramatic expansion of genetic counselling and prenatal testing into clinical routine in the 1970s. With prenatal diagnosis as its ‘technological handmaiden’, genetic counselling developed into a practice for defining and handling the various risks associated with the birth of a handicapped child (Gaudillière, 2011; Stern, 2012, p. 168).

While postwar transformations in medical genetics are well documented, historians have yet fully to recognize parallel concerns about the environmental causes of birth defects. Although clinical observations and experimental studies of environmental factors in development proliferated before World War Two, British epidemiological interest in congenital malformations originated largely in the postwar reconstructive ideals of social medicine (Porter, 1997). Dugald Baird, director of the Aberdeen Social Obstetric Unit led a range of studies indicating that high fetal mortality correlated with poor social conditions (Davis, 2009). Baird and his colleagues argued that eliminating poverty and improving women’s health and nutrition during growth and adolescence as well as pregnancy would lower the incidence of stillbirths (Baird, 1974; Duncan, Baird, & Thomson, 1952). Declining mortality from infectious disease made congenital malformations more visible as causes of stillbirth and infant and childhood mortality. Thomas McKeown and collaborators at the Department of Social Medicine, University of Birmingham, explored environmental influences such as season of birth, maternal age and birth rank and showed how these were related to socio-economic status (McKeown & Record, 1951; Record & McKeown, 1949).

This work initiated the first population-based continuous register of malformations, which stimulated an international network of similar registers (Leck, 1996). Attention to the surveillance of birth defects had intensified enormously in Britain during the 1960s in response to thalidomide and epidemics of German measles. In 1964 the Ministry of Health instituted a formal system of registering congenital malformations, with the aim of establishing typical seasonal and regional variations in incidence, and of warning quickly of any unusual increases (Anon, 1964a, pp. 71–72). These registries revealed especially high rates of NTDs in particular regions of Britain: north-west England, Northern Ireland and the coal-mining valleys of South Wales. Much subsequent research was predicated on the assumption that the prevalence of NTD births was highest in the ‘underprivileged areas of poverty’ (Nevin, 2004; Stocks, 1970). In some regions, incidence was as high as four in every 1000 births (Anon, 1968a).

British epidemiologists’ preoccupation with NTDs developed in tandem with intense public concern about the medical, social and ethical dilemmas associated with innovations in the surgical treatment of spina bifida. Before 1960, the survival rate for all forms of spina bifida was 10% to 12%. Paediatric surgeons typically postponed treatment until the infant reached the age of two, believing that only the strongest would survive that long. However, many surgeons increasingly argued that early intervention significantly improved the prognosis of spina bifida babies. Supported by the increasingly widespread provision of specialist neonatal care (Christie & Tansey, 2001; McAdams, 2008), most paediatric units in the UK by the mid-1960s operated within 12 to 48 hours of birth on all infants who did not have other defects incompatible with life (Pruitt, 2012; Sharrard, Zachary, & Lorber, 1967). Although this shift in practice requires further historical analysis, contemporaries perceived a ‘spectacular increase in the number of newborns [with spina bifida] admitted to the few centres in Great Britain which are equipped to deal with them’ (Anon, 1964c). By the mid-1960s, NTDs had become an ‘urgent medical, social, epidemiological, and aetiological problem’ (Laurence, Carter, & David, 1968). Two new charities, the Association for Spina Bifida and Hydrocephalus (ASBAH) and the Spina Bifida Trust, responded to mounting concern by fundraising for medical research and to support families and individuals affected by these conditions. Action Research for the Crippled Child (ARCC), one of the major charities supporting medical research in the UK, also shifted its funding priorities from polio to congenital malformations in the mid-1960s (Action Medical Research, 2012).

The increased visibility of spina bifida in the 1960s precipitated discussions about the ‘burden’ of the physically handicapped on not only individual families, but also the public purse. Even as disability rights activists began to campaign for better access to education and employment (Millar, 2010), many commentators worried about the greater demands of ‘long-term survivors’ of spina bifida for specialist medical care and schooling. According to Gerald Leach, science correspondent for the *Observer* newspaper, resource allocation to cope with the ‘rising tide of chronic handicap’ urgently needed debate (1970, p. 182). For McKeown, because the ‘malformed child’ represented an ‘intolerable burden on the medical and social services’, it was time to ‘establish national priorities in the use of medical manpower’ (1967, pp. 1223–1224). Other medical practitioners more bluntly—and provocatively—argued that ‘the spina bifida baby is a mistake of nature not equipped to survive … the death costs nothing; the life costs not only money but the pre-emption of precious medical, nursing, social and educational resources’ (Slater, 1971, p. 735). Although many paediatricians began to endorse the view that ‘society needed to take a more selective approach’ to early surgical intervention (Anon, 1972), the alleged ‘neonatal euthanasia’ of spina bifida babies had become increasingly controversial (Anon, 1974).^6^

New emphasis on the ‘prevention of handicap’ as a solution to these social and ethical dilemmas during the 1970s was part of a broader trend in public health in Britain. The policy focus on ‘prevention’ as a matter of personal responsibility originated in the postwar period as epidemiological attention shifted from infectious to chronic diseases as the major cause of ill health and mortality. But the notion of prevention as individual responsibility was carried further in the 1970s, especially following the influential 1976 Department of Health and Social Security policy report, *Prevention and Health: Everybody’s Business* (Berridge, 1999, pp. 87–88; DHSS, 1976). *Prevention and Health, Reducing the Risk: Safer Pregnancy and Childbirth* (DHSS, 1977), the first of a series of follow-up discussion papers, sought to determine how far the new philosophy of preventive medicine could be applied to pregnancy and childbirth. Of particular concern was the need to reduce the prevalence of handicap, claimed to impose ‘great burdens on our society’. Because of the ‘substantial costs’ of treating spina bifida in particular, a programme to prevent the birth of children with this disability promised almost immediate savings (DHSS, 1977, pp. 7, 42, 48). In practice, this meant supporting the extension of new prenatal diagnostic technologies into comprehensive screening, and offering women the choice to terminate an abnormal pregnancy. Research in the 1970s led by the Edinburgh geneticist David Brock established that elevated levels of alpha-fetoprotein (AFP) in amniotic fluid and later, in pregnant women’s blood indicated that the fetus was at risk of NTD led rapidly to routine testing for this marker (Brock & Hughes-Davis, 1974; Brock & Sutcliffe, 1972). The cost-benefit rationale remained omnipresent in discussions around the introduction of these technologies as medical care was increasingly constrained by budgetary pressures in the 1970s and 1980s (Gagen & Bishop, 2007; Stern, 2012, pp. 161–164).

However, the rapid diffusion of amniocentesis and AFP-screening in the late-1970s was not uncontested, and ‘prevention’ did not only mean abortion. The Royal College of Obstetricians and Gynaecologists initially resisted the generalizing of prenatal screening on the grounds that the technology was not foolproof; members feared the prospect of terminating healthy pregnancies following false positives (Yanchinski, 1978). Other clinicians worried that health service planners’ use of prenatal diagnosis and selective termination was overshadowing ‘research into the causes and true prevention of birth defects’ (Harris, 1980, p. 1199). This included studies into the teratogenic properties of new drugs, infectious diseases, synthetic hormones, environmental pollutants, alcohol and smoking. In the period after thalidomide, the popular and medical press frequently reported new theories about the alleged causes of fetal deformities (Renwick, 1972, p. 279). As an editorial in the *New Scientist* observed, ‘the thalidomide tragedy … transformed teratology from an obscure laboratory game into a valuable clinical science’ (Anon, 1973). While many theories were discounted as quickly as they were proposed, the possibility that vitamin deficiencies increased the risk of NTDs remained persuasive.

In the 1960s, the paediatrician Richard Smithells collaborated with local obstetricians in researching folate metabolism in reproduction in Liverpool, noted for its high NTD prevalence. Smithells had set up a congenital abnormalities register and genetic counselling service for Liverpool in 1960 (Smithells, 1962) and became the leading British expert on thalidomide diagnostics. Offering medical testimony to support the plaintiffs’ case against the drug’s UK distributor, he was subsequently involved with the trust set up to provide ‘relief and assistance’ to thalidomide children (Schorah, 2009). Smithells then started a series of small-scale studies that suggested a ‘significant relationship’ between a positive FIGlu test for folate deficiency and fetal malformation (Hibbard & Smithells, 1965). With funding from the ARCC and the Children’s Research Fund, a Lancashire-based charity established in the wake of thalidomide, Smithells built a research group at the University of Leeds to investigate the broader role of drugs and nutrition in pregnancy and birth defects (Smithells, Sheppard, & Schorah, 1976; Smithells et al., 1977). Motivated by the view that the diagnosis and termination of a fetus with spina bifida was a ‘poor substitute for prevention’ (Anon, 1992, p. 1062), Smithells proposed to subject the folate hypothesis to the scrutiny of a clinical trial. What if a woman ‘at risk’ of having a child with an NTD could be given supplementary vitamins *before* her next pregnancy? Thalidomide had revealed that drugs could harm the developing fetus, but could a pharmaceutical product be shown to *prevent* birth defects?

Such an experiment was plausible only in a British medical and social landscape transformed by thalidomide and the legalization of abortion. The rapid expansion of prenatal diagnosis in the ten years after the Abortion Act consolidated the genetic counselling clinics from which women with a history of an NTD pregnancy could be recruited. Yet the possibility of a trial relied upon not only the willingness of what the tabloid press came to call ‘heartbreak mums’ to be recruited, but also the construction of spina bifida as an ‘urgent’ problem for the medical profession, charities and society at large. Launching a clinical trial in 1977, Smithells would transform folic acid from a routine prenatal vitamin to reduce the risk of anaemia into an experimental drug for preventing NTDs in ‘high-risk mothers’.

## 4 Trialing pre-conceptional vitamin supplements and ‘high-risk mothers’

Smithells’ team at Leeds, with collaborators in Manchester, Chester and London, succeeded in recruiting 137 ‘study mothers’ into a trial of ‘Pregnavite Forte F’, a multivitamin containing folic acid manufactured by the British pharmaceutical company Beecham and available only on prescription. Each woman agreed to take three tablets a day for no fewer than 28 days before conception, up until the date of the second missed period. A second group of 187 women who were already pregnant when referred to the study or who declined to take supplements were used as controls. The preliminary findings, published in the *Lancet* in 1980, suggested that the NTD recurrence rate in supplemented mothers was around half that of the control group (Smithells et al., 1980). Smithells framed this research as contributing to the ‘primary prevention’ of malformations, as opposed to ‘secondary prevention’ through prenatal screening. The promise of reducing not only birth incidence of neural-tube defects but also the need for selective termination made this an ‘exciting finding’ in the context of continued debate over abortion (Anon, 1980; Davis, 1985).

In the weeks and months following its publication, Smithells’ report attracted a lively correspondence in the *Lancet*. Critics rounded on the design of the trial; in particular, the ‘self-selecting’ method of recruiting participants and the use of a ‘blunderbuss’ multivitamin. Smithells and his colleagues were accused of inducing women to ‘self-administer large quantities of multivitamins’ on the basis of a trial that was not properly controlled, and failing to resolve uncertainty over the causal relationship between folic acid and NTDs.^7^ Despite these doubts, the practical response of many clinicians was to behave as if there was a link. Within months, genetic counsellors were ‘already recommending [Pregnavite] to mothers with a history of NTD pregnancies’ (MRC file note, National Archives FD23/5150). Many clinicians were prepared to accept Smithells’ results as imperfect, but adequate in practical terms. For epidemiologists, however, the design of the trial could not support valid conclusions of cause and effect and—potentially worse—generated new risks over the actions of vitamins that were ‘imperfectly understood’.^8^

Long-term prenatal multivitamin supplementation raised the spectre of the synthetic estrogen diethylstilbestrol (DES), recently found to have caused genital malformations and cervical cancer in the daughters of pregnant women to whom it was prescribed to prevent miscarriage (Chalmers & Sacks, 1982; Langston, 2010). To Smithells’ critics, the poorly designed trial rendered Pregnavite ‘of such uncertain efficacy and safety that it should be regarded as a subject of clinical research rather than accepted medical practice’ (MRC press release, National Archives FD23/5152). While acknowledging the need for more research, Smithells and his supporters employed the language of ‘promise’ and ‘guarded optimism’ (MRC file, National Archives FD23/5150). Those for whom a double-blind, placebo-controlled trial was the bottom line spoke of continued ‘uncertainty’ and new ‘risks’. Even as medical practitioners prescribed vitamins to high-risk mothers, the debate over the credibility of existing data intensified. The decision of the Medical Research Council, at the request of the UK government, to initiate a randomized controlled trial of pre-conceptional vitamin supplementation marked the start of a more public phase in what was later claimed as ‘one of the great medical controversies of the twentieth century’ (Stone, 1991).

The randomized clinical trial (RCT) had, by around 1970, gained a form of scientific and bureaucratic orthodoxy as the ‘gold standard’ of postwar biomedical research (Goodman, McElligott, & Marks, 2003; Löwy, 1996, pp. 36–83; Marks, 1997). The epistemological success of the multi-centre RCT in Britain was largely the achievement of the MRC, whose pioneering trials of the anti-tuberculosis drug streptomycin in the late 1940s laid the parameters for biomedical research during a period of increased centralization of health policy and planning. Supporting the new role of the state as ‘scientific entrepreneur’, the MRC pursued RCTs as ‘a means of unifying a research landscape that was characterized by localism’ (Valier & Timmerman, 2008, p. 494). Research into the role of folate deficiency in reproduction had certainly been located in heterogeneous disciplinary traditions that served various academic, clinical, and charitable agendas. The suggestion that pre-conceptional vitamins could reduce NTD prevalence by half now had such significant health policy implications as to push the MRC to become involved. Should the state fund the provision of folic acid supplements, or a proprietary multivitamin at more than 35 times the cost?^9^ How, above all, to reach women before they became pregnant? Official recognition of the need for a multi-centre RCT not only reflected the view that previous studies, with their diverse sources of funding and research protocols, were too inconclusive to inform such major policy decisions as the national healthcare budget contracted under the Conservative government of Margaret Thatcher. It also reinforced the position of the MRC as the pre-eminent producer of medical knowledge and arbiter of best practice in the UK.

Yet even as the MRC and other organizations consolidated the status of the RCT, controversies over human experimentation through the late 1960s and 1970s began to change the landscape of clinical research (Cooter, 2000, pp. 458–459). In response to mounting critiques of risky clinical experimentation without informed consent, British hospitals set up specific committees to oversee the ethics of medical research. In 1984, one year into the MRC vitamin study, the Department of Health established Local Research Ethics Committees with the aim of enhancing protections for subjects based on the principles of the Helsinki Declaration of 1964 of the World Medical Association (WMA) (Hazelgrove, 2002; Hedgecoe, 2009). Amendments to the Helsinki code at the WMA assembly in 1975, the first of eight subsequent revisions to date, placed particular emphasis on tightening definitions of informed consent (Faulder, 1985, pp. 48–49). MRC policy on informed consent in the 1980s had been laid down in a 1964 statement on ‘Responsibility in Investigations on Human Subjects’, long regarded as the authoritative guide for clinical research in the UK. This distinguished between procedures of ‘benefit to the individual’ and those ‘undertaken either on patients or on healthy subjects solely for the purpose of contributing to medical knowledge’. Investigators were required to obtain ‘explicit’ written consent in the latter case but not the former, since experimental treatments were assumed to ‘fall within the ambit of patient-care’ (MRC, 1964, p. 178).

Through the 1980s and 1990s, new procedures for the oversight of the profession emerged alongside rising appeals for lay involvement in the administration of clinical research in the form of patient activists and advocates, consumer groups and the independent discipline of bioethics (Dawes, 2012; Epstein, 1996; Wilson, 2012). By the 1970s the mass media was becoming ever more central to establishing the dimensions of scientific and medical controversies, including shaping both lay and professional perceptions of certainty, uncertainty and ethical practice in clinical trials (Berridge & Loughlin, 2005; Epstein, 1996, p. 22). The MRC trial launched just as the long-running public controversy over claims that large doses of vitamin C could cure cancer was about to reach its climax (Richards, 1991). Meanwhile, tighter regulation of drugs testing in both the UK and the US, along with increasing demands for larger trials to ensure greater statistical confidence in the results encouraged the ‘outsourcing’ of clinical research, especially to middle- and low-income countries from the 1980s (Petryna, 2009). Symptoms of all these trends can be seen in the design of and response to the MRC vitamin study.

The MRC steering committee established to design and coordinate the trial first convened in February 1981. The study was finally launched late in 1983, initially involving 17 participating centres in the UK (MRC Vitamin Study Research Group, 1991). From the outset the MRC was sensitive to the ethical implications of enrolling women who had previously had an NTD baby and were planning another pregnancy into a controlled trial. This would mean randomly allocating subjects into one of three control groups: the first to take a daily Pregnavite multivitamin, the second a 5-milligram dose of folic acid, and the third a placebo. Where mandatory AFP testing and amniocentesis indicated a fetal NTD, women would be offered an abortion. The steering committee initially spoke of recruiting a minimum of 2000 and as many as 5000 participants over three years, but asserted that the trial would be terminated as soon as there was statistically significant evidence of a ‘very marked’ reduction in NTDs in either arm of the study (National Archives, MRC press release; Report to the Systems Board FD23/5152). Criticisms of the proposals surfaced immediately, with some experts invited to comment on the trial design arguing that it would be ‘unacceptable to withhold a harmless, and possibly beneficial, treatment from women who had presented for treatment.’^10^ John Davis, the Cambridge professor of paediatrics appointed to chair the MRC study group, was soon ‘bombarded’ with letters from medical colleagues critical of plans to include a placebo group (Davis, 1982d).

The debate spilled into public view early in 1982 as journalists picked up on expert disagreement over the feasibility and ethics of a placebo-controlled trial. The Oxford geneticist John Edwards’ proclamation on BBC television’s flagship science programme ‘Tomorrow’s World’ that the MRC trial was unethical sparked a ‘media storm’ that would delay the launch of the trial by over a year (Edwards, 1982a, 1982b). The lay press were universally hostile to the trial, presenting Smithells’ ‘remarkable’ findings as having settled the issue (King, 1982). Newspaper copy denounced the MRC for ‘deceiving’ thousands of women into what amounted to a ‘cruel game of Russian Roulette’ (Toulson, 1982). The steering committee sought to manage the controversy by warning editors in both the lay and scientific press that ‘public and emotive debate … [made] better journalism rather than better medicine’ (Davis, 1982b). But the debate had already moved far beyond their control even when an opposition politician demanded the MRC abandon a ‘miserable and unworthy piece of medical research’ (Medical Correspondent, 1982). By December 1982, public controversy over the placebo left the MRC with a ‘debacle’ that threatened the likelihood of recruiting sufficient numbers of research subjects (Davis, 1982c; Middlemiss, 1983).

That the media should, from the point of view of the MRC, ‘trivialise’ scientific debate over the conclusiveness of Smithells’ results and the necessity of a placebo, is not surprising.^11^ Yet the MRC had little, if any, previous experience defending the design of a clinical trial in public. The pre-conceptional vitamin study set a precedent for well-known controversies over placebo-controlled trials of the AIDS drug AZT later in the 1980s (Berridge, 1996, p. 235; Campbell, 1988; Epstein, 1996). The major consequence of journalists’ attention was that it hardened many experts’ private concerns about the feasibility of the trial into overt criticism of its ethics. As Edwards put it, ‘now that [Smithells’ evidence] has been accepted by ‘Tomorrow’s World’ any criticism must be clear and open’ (Edwards, 1982b).

As for Smithells’ results, disciplinary factors played a central role in dictating positions for and against the trial. Epidemiologists defended the placebo-controlled trial as a ‘social responsibility’ and the only way to achieve scientific certainty (Doll, 1982). The organizers argued that participants in the trial would be willing to ‘pay the price’ of an abortion ‘in order to contribute certain knowledge where at the moment only opinion prevails’ (Davis, 1982a). The most stringent opposition came from clinicians concerned that they could not subject women to the risk of a placebo without violating ordinary responsibilities of patient care, not least because vitamins, including folic acid, were already so widely recommended in pregnancy. Doctors critical of the trial typically invoked personal moral judgment, advising patients that that they ‘would not wish a member of [their] own family to forgo the possible advantages of supplementation’ (Davis, 1981). Disagreement hinged above all on whether women recruited to the trial should be considered pure research subjects or patients, a distinction that would in turn determine the necessity of obtaining explicit informed consent (Davis, 1982c). Lack of clarity on this issue provoked concern that doctors who participated in the study could be liable to charges of negligence (Leahy Taylor, 1983).^12^ In sum, even as the inclusion of a placebo group promised to give the MRC study the biomedical credibility that Smithells’ research lacked, it threatened to undermine the credibility of medicine as a healing profession (Faulder, 1985, pp. 92–93). The controversy pitted what one opponent of the MRC trial termed ‘common sense against the purism of some academic epidemiologists’ (Renwick, 1982).

A year into the MRC study, participating centres had managed to recruit a total of just 132 women, far short of the minimum 2000 target (Abel, 1984). When the trial was ultimately concluded it was widely assumed that the ‘media storm’ of the early 1980s ‘slowed recruitment to such an extent that it took eight years to accumulate enough participating women and enough pregnancies’ for a ‘conclusive’ result. Although the possibility of recruiting subjects from outside Britain had been mooted from the start, the study ultimately relied upon 16 centres in Australia, France, Israel, Hungary and the then USSR. More volunteers were enrolled at the seven centres in Hungary than the UK, despite a similar NTD rate (Anon., 1991, p. 153). Public controversy around the ethics of the trial forced those with a stake in the research into polarized positions on the reliability and implications of Smithells’ findings: as being either for ‘social responsibility’ or opposed to ‘denying’ ‘high-risk mothers’ and their fetuses the vitamin supplements they required. The extension of the debate beyond the medical press not only raised the stakes for the researchers, but also brought a range of new participants’ differing perceptions of risk, nutrition, motherhood and disability into conflict.

## 5 Constructing pre-conceptional nutrition

The MRC vitamin study group announced the results of the trial in 1991, claiming a ‘72% protective effect’ for folic acid in at-risk pregnancies (MRC Vitamin Study Research Group, 1991). An editorial in the *Lancet* declared the debate over the connection between folate deficiency and NTDs ‘resolved at last’, while promoters of ‘evidence based medicine’ would commend the study as a model of good practice (Anon, 1991; Oakley, 1990; Chalmers et al., 1993). Such acclaim both disguised earlier disagreement and failed to anticipate new debate over the implications of the study’s findings. Above all, 95% of NTDs were first-occurrence; the question remained as to whether folic acid supplements would ‘protect’ women with no previous history of an NTD pregnancy (Czeizel & Dudás, 1992). Moreover, claims that the statistical rigour of the trial produced definitive answers overlooked the vital role of groups other than MRC epidemiologists in tipping the balance of certainty and uncertainty. The new identity of folic acid as a risk-reducing drug depended on the agency of non-experts.

First, the media, as discussed above, played a major part in the controversy, not merely communicating expert disagreement, but also shaping both professional and lay perceptions of risk and uncertainty. Mass media debate required that protagonists adopt a clear position on the ‘ethics’ of the trial and, thus, the efficacy of pre-conceptional vitamins in reducing the risk of NTDs. Medical researchers and practitioners used the media to declare their support or opposition to the trial and journalists intervened directly, too. The feminist health rights activist and journalist Carolyn Faulder, for instance, framed the trial as part of a patient-consumer struggle for informed consent. Condemning the MRC as an ‘implacable stony-faced god of numbers’, Faulder claimed that that vulnerable women were effectively being deceived into ‘sacrific[ing]’ their children for the sake of statistical certainty (1985, p. 92). When the MRC sought to dissuade critics of the trial from expressing their concerns in public, *Nature* ran an intentionally ‘spectacular’ blank space on the editorial pages in protest at a blatant attempt to stifle professional debate (Veitch, 1982a).^13^ More generally, newspapers produced a whole language around the trial distinct from the expert discourse. ‘Study mothers’ were recast as ‘guinea pigs’ and ‘heartbreak mums’; ‘affected pregnancies’ became ‘tragic babes’; multivitamins counted as ‘special pills’ and placebos were ‘fake ones’ (Ferriman, 1982; King, 1982; Roxan, 1982; Toulson, 1982; Veitch, 1982b).

Second, industry had a vested interest in the design and interpretation of the trial. Beecham, who supplied Smithells with the ‘blunderbuss’ multivitamin criticized by epidemiologists, withdrew from the MRC study in 1981, unwilling either to manufacture supplements other than Pregnavite or to provide them free of charge (MRC file note, National Archives FD23/5152). Beecham’s medical director had also raised concerns about the ethics of the placebo group. The MRC took this stance as a subterfuge, recognizing Beecham’s obvious interest in having the assumed effectiveness of ‘their mixture’ demonstrated by a major clinical trial and the likelihood that the supplements would be ultimately recommended to ‘the entire population of women at risk of becoming pregnant’ (Davis, 1982a). Beecham’s announcement in 1982 that it had withdrawn from the trial because ‘it believed the Smithells work was conclusive’ coincided with the most intense phase of the media controversy (Bryan, 1982b). Within months of the publication of the vitamin study in 1991, food manufacturers announced plans to fortify proprietary products with folic acid. The Kellogg Company, for instance, launched a new marketing campaign targeted at ‘women of childbearing age’, drawing on the MRC study as proof that ‘babies benefit from a healthy breakfast even before they’re conceived’.^14^ At precisely the time advertisers used fetal images to sell family cars (Taylor, 1992), invoking the benefits of folic acid to ‘babies’ even before pregnancy helped market breakfast cereal.^15^ In the early 1990s, folic acid fortification became a key element in the branding of both proprietary multivitamins and processed foods, preceding official public health initiatives by some four years (Nestle, 2007, pp. 303–304).^16^ As of 2013, pharmaceutical companies remain the main corporate sponsors of folic acid awareness campaigns, on which they rely to promote other ‘pregnancy vitamin’ combinations.^17^

Third, charities, patient advocates and consumer groups were deeply involved in the controversy from the start, actively influencing the design of the trial. Most directly, the cooperation of the Association for Spina Bifida and Hydrocephalus (ASBAH) was crucial to the recruitment of research subjects. Preconditions for ASBAH endorsement were that participants would be ‘adequately briefed’ before giving their consent and that even the placebo group would receive a mineral supplement containing iron and calcium rather than a ‘dummy pill’ (Bryan, 1982a; Wynn, 1982).^18^ ASBAH was part of a coalition of women’s health and disability organizations campaigning for the reform of maternity care, including the National Childbirth Trust (NCT), the Association for the Improvement of Maternity Services, the Foundation for Education and Research into Childbearing and the Maternity Alliance. Although these ‘consumer’ groups differed in their objectives and methods, by the early 1980s they came to focus much of their campaigning around reducing maternal poverty and preventing handicap (Oakley, 1984, pp. 236–249; Wynn & Wynn, 1979). While the MRC won the support of ASBAH by revising the trial protocols, the maternity groups remained critical, arguing that the study ignored the real problem of dietary deficiency among socio-economically disadvantaged women (National Childbirth Trust, 1983). The Maternity Alliance, in particular, sought to highlight the wider issue of maternal poverty, agitating against the Thatcher Government’s cuts to the maternity budget and for the provision of free vitamins to pregnant women (Phillips, 1984, 1986). This campaign framed broader debate over the importance of pre-conceptional nutrition and the prevention of disability.

Fourth, media coverage of the trial mobilized individuals and groups sympathetic to the agendas of the overlapping anti-abortion and disability ‘right-to-life’ movements. Pro-life groups’ opposition to the trial built on recent campaigns against prenatal screening and efforts to instigate criminal proceedings against medical practitioners for the selective non-treatment of newborns with disabilities (Read & Clements, 2004). The then largest ‘pro-life’ organization in the UK, the Society for the Protection of the Unborn Child (SPUC) led the anti-abortion response from summer 1983, coordinating pickets of branches of Boots chemists, whose pharmaceutical division had supplied the multivitamin and placebo pills.^19^ Leaflets handed out at the pickets demanded the MRC ‘stop these immoral trials … condemned as immoral and repugnant by many organizations and doctors throughout the country’. SPUC claimed the ‘Smithells regimen’ was ‘widely recognized throughout the world’, urging the public to write to their MPs and to ‘support spina bifida people’ by boycotting Boots and complaining to their local stores.^20^ Although SPUC led protests against the trial, anti-abortion groups consistently framed ‘vitamin prophylaxis’ as more ‘morally acceptable’ than both terminating pregnancies for fetal abnormality and research on human embryos.^21^ Pro-life MPs invoked this claim in parliamentary debates around the almost successful Unborn Children (Protection) Bill in 1985 and the Human Fertilisation and Embryology Act in 1990, reinforcing the view that certainty in pre-conceptional vitamin supplementation had already been achieved (Evans & McLaren, 1985; Hansard, 1990: Winterton).^22^

Industry, consumer groups, and pro-life activists were all invested, for different reasons, in promoting ‘better’ nutrition before and during pregnancy. Controversy around the MRC trial drew these heterogeneous actors, as well as research scientists, clinicians and journalists, into a single ‘arena of concern and action’ (Clarke, 1998, p. 16); all were heavily involved in the development and marketing of pre-conceptional vitamin supplements. The crucial role of these groups in the public construction of certainty and uncertainty is essential for understanding the emerging identity of folic acid as a risk-reducing drug. Their involvement both generalized and popularized discussions on the relationship between nutritional deficiencies and NTDs, turning pre-conception vitamin supplementation into a risk-reducing strategy not only for ‘high-risk mothers’ but for all women planning a pregnancy. By organizing for and against the trial, moreover, the healthcare industry, the media and patient advocates, also helped to reify the concept of ‘pre-pregnancy’ as a legitimate focus of medical and social intervention.

Immediately following the publication of the MRC study, the UK Chief Medical Officer issued a circular recommending that all women of childbearing age significantly increase their daily folate intake (Acheson, 1991). In the same year, the parliamentary health committee confirmed that the expansion of ‘preconception care’ would be a key objective of maternity services reform (Health Committee, 1991). Although increasingly offered within NHS hospitals by the mid-1980s, ‘pre-conception clinics’ originated as a private healthcare initiative. Largely associated with Foresight, a charity established in 1979 for the ‘promotion of pre-conceptual care’, the clinics focused on preparing women for pregnancy as a means of ‘preventing handicap’ (Oakley, 1984, p. 291). Promoted in the media and by maternity consumer groups through the 1980s and early 1990s, Foresight has campaigned to raise awareness of ‘risks to the foetus of nutritional deficiencies’, as well as marketing their branded vitamin supplements, to help couples ‘build a better baby’ (Cochrane, 1983). This innovation provoked mixed responses. Some in the medical profession saw pre-conception care as a means of helping obstetricians to ‘reduce further fetal wastage and the incidence of malformation’ (Anon, 1981; Smithells, 1984, p. 433); for others, it merely dragged ‘the medicalisation of pregnancy and birth backwards to cover conception as well’ (Anon, 1985, p. 1047). British feminists took pre-pregnancy care as emblematic of the ‘moral climate’ of the 1980s, coinciding with attempts by the ‘New Right’ to reduce the legal limit of abortion, and extending social expectations around the responsibilities of motherhood to include not only pregnancy, but also the period before conception (McNeil, 1991, p. 151). Pre-conception care has nevertheless become an international movement, with the promotion of folic acid supplementation a key objective (Casper & Moore, 2009, pp. 67–72).

## 6 Conclusion

Originally prescribed to pregnant women in order to reduce the risk of anaemia, folic acid was by the late twentieth century a technology of ‘pre-pregnancy’ for preventing neural tube defects, the most common congenital malformations. As one component of the Pregnavite multivitamin, it was an ‘experimental drug’ for women at ‘high risk’ of bearing a child with an NTD. By the mid-1990s, health education materials recommended folic acid supplements to ‘all women who may bear children’. If epidemiology in the tradition of social medicine encouraged NTDs to be understood in relation to social problems of poverty and malnutrition, by the end of the century they were more likely to be attributed to individual women’s failure to respond appropriately to expert guidance. New guidelines about preconceptional and prenatal nutrition came as medical research and related advice concerning pregnancy in general, including the risks maternal drinking, smoking and drug taking posed to the fetus, proliferated in the 1980s and 1990s (Daniels, 1993). At that time, a few health activists and journalists observed that the extreme focus on expectant women’s personal accountability for birth outcomes not only served to intensify both their anxieties during pregnancy and feelings of guilt about bearing a disabled baby, but also distracted attention from debate over the socioeconomic determinants of maternal and infant health (Kent, 1986). Feminist scholars have since widely critiqued the “privatization of risk” in pregnancy and the exaggerated emphasis on maternal responsibility for unborn or even yet-to-be conceived fetuses that dietary guidelines have encouraged (Lupton, 2012; Markens, Browner, & Press, 1997). But demands for expectant women to engage in risk-aversive behaviours, this article suggests, did not result straightforwardly from medical professionals’ aspirations to expand the scope of obstetric control over pregnancy.

The trajectory of folic acid from experimental drug to necessary pre-conceptional supplement was negotiated over several decades by the medical researchers, healthcare industry, the media, consumer groups and activists. Reflecting on the controversy over the MRC study in 1984, the sociologist Ann Oakley observed that ‘diet is something everyone is an expert on, and the idea that malformed fetuses can be prevented by a healthy diet … [has] a long history and a commonsense appeal’ (1984, p. 290). Activists’ interest in nutrition, especially of mothers and infants, both followed and supported a swathe of research across the medical and social sciences during the 1970s and 1980s. Debates over folic acid need to be placed more fully in relation to the wider politicization of food and nutrition in this period (Nestle, 2007; Scrinis, 2013). Medical research into folic acid was, perhaps above all, stimulated by the emergence of spina bifida as a political concern in Britain around 1970. This transformation remains poorly documented, and we need more nuanced histories of the connections between medical innovations, public health policies and ‘disability rights’ activism in a period of rapid change. By 1991 the context was entirely different; the birth prevalence of NTDs had declined significantly and spina bifida was far less visible than it had been even ten years earlier.^23^ The conclusion of the MRC study was nevertheless an important watershed in that it directly informed health policies in the UK and around the world. Yet its publication merely opened up a new phase of controversy.

The 1996 ruling by the United States Food and Drug Administration to implement a national folic acid fortification policy was widely hailed as ‘a true victory in the battle to enhance the health of future generations of babies’ (Junod, 2001; Semba, 2006, p. 176). Yet consumer groups and food campaigners accused the FDA of mass-medicating the American population without thoroughly understanding the long-term effects of folic acid consumption and working in the interests of manufacturers of often expensive processed foods. Similar arguments have been raised in opposition to mandatory fortification in the United Kingdom (Blythman, 2007, 2008; Nestle, 2007, pp. 303–304; Woffinden, 1998). As of 2009, over 65 countries worldwide had followed the US lead. Numerous scientific advisory bodies have recommended mandatory folic acid fortification to successive UK governments, but experts remain locked in debate over the risks and benefits (Wald & Oakley, 2007). Advocates of fortification have consistently complained that, because more than half of all pregnancies are unplanned, the educational campaigns hitherto favoured in the UK and other European countries are inadequate (Smith, Davies, & Davies, 1994; Sutcliffe, Schorah, Perry, & Wild, 1993). Opponents have argued that high folic acid intake may both mask the signs of anaemia in diets deficient in vitamin B12 and accelerate the development of certain cancers (Hubner, Houlston, & Muir, 2007). Scientific disagreement, in addition to perceived governmental recalcitrance, has made folic acid enormously contentious in Britain over the last two decades.

The tumultuous trajectory of pre-conceptional folic acid supplementation invites comparisons with other postwar medical interventions, particularly those concerned with reducing the risk of congenital and genetic disability and disease. Further research might also ask to what extent measures to encourage ‘pre-pregnant’ women to consume supplements have followed precedents set by rubella vaccination (Reagan, 2010), newborn screening for phenylketonuria (Lindee, 2005; Paul, 1998, pp. 173–186) and campaigns around smoking in pregnancy (Berridge & Loughlin, 2008) and fetal alcohol syndrome (Armstrong, 2003; Golden, 2005). Like prenatal diagnosis, these innovations have contributed to the inexorable rise of the fetus in medical practice and public debate. Continuing disagreement among health policy makers over folic acid also open up further questions about varied public responses and assessments of success or failure in different national contexts. In particular, the ways in which the fate of pre-conceptional vitamin supplementation was tied to other modalities of reducing birth defects—specifically, prenatal testing and selective termination—and controversies around them demand further study. Like many recent public health innovations, folic acid was scaled up from an individual treatment for selected, high-risk individuals into a means of accomplishing population health goals. As public health experts continue to debate the risks and benefits, it nonetheless remains an experiment-in-progress.

## Footnotes

1 According to the UK National Health Service online Information Service for Parents, ‘Start 4 Life’: http://www.nhs.uk/start4life/Pages/vitamin-supplements-folic-acid-pregnant.aspx (accessed 15 December 2012).

2 Robert Aronowitz has called for greater historical sensitivity to the “generation-specific and contingent influences on the appearance and identity of risks, diagnoses and prevention practices” (2006, p. 156).

3 Quotations from advertisements for Stork and Bemax in the *Observer*, 24 April 1938: 28 and the *Guardian*, 7 May 1953, p. 4.

4 ‘The first pinch cost a fortune’, Advertisement for Lederle, *Life*, 17 November 1952, p. 72.

5 See advertisement for Marmite Yeast Extract, in the *Journal of Tropical Pediatrics* 13, 1967, frontmatter.

6 This debate was crucial to arguments that external oversight would make medicine more accountable to the public, and in the emergence of bioethics in Britain from the late 1970s: Wilson (2012).

7 *Lancet*, 22 March 1980, pp. 647–648; 17 May 1980, pp. 1061–1062; 14 June 1980, p. 1302.

8 Clinicians and epidemiologists were similarly divided over the relationship between low-level radiation and childhood leukaemia in the 1950s: Dry (2006).

9 In 1991, a year’s supply of folic acid supplements cost £1.24 per person, compared with £44 for the Pregnavite Forte F multivitamin: Kingsland (1991).

10 Multivitamins and neural tube defects: draft minutes of an ad hoc meeting, 15 December 1980 (National Archives, FD23/5150).

11 Letter from *Daily Mail* medical correspondent Paul Crosbie to Sir James Gowans, secretary of the MRC, 9 December 1982 (National Archives, FD23/5154).

12 This distinction had led the MRC, a decade earlier, to reject an application for support for clinical research into IVF: Johnson, Franklin, Cottingham, & Hopwood (2010).

13 *Nature*, 25 November 1982, p. 302.

14 See Kellogg advertisement for fortified cornflakes in *The Guardian*, 18 December 1991, p. 3.

15 The anthropologist Janelle Taylor has suggested that the use of fetal images in advertising is ‘designed to channel political passions’ around abortion into consumer behaviour: (2008, p. 3).

16 Industry has used clinical trials more generally as a means of promoting specific products and building brand loyalty: Petryna (2009, p. 96).

17 For instance, Bayer HealthCare and Vitabiotics, both manufacturers of proprietary pregnancy vitamins, sponsor the most recent folic acid education campaign run by ASBAH (renamed SHINE in 2011): http://www.gofolic.co.uk/, accessed 10 February 2013.

18 Women were allocated at random to one of four supplementation groups, the supplements containing folic acid, multivitamins, both, or neither. All contained both iron and calcium.

19 *Catholic Herald*, 12 August 1983, p. 3. On SPUC, founded in 1967 to campaign for reform, and ultimately repeal, of the Abortion Act, see Marsh & Chambers (1981).

20 All quotes from SPUC leaflet (1983).

21 Gray (1983); *Catholic Herald*, 18 March 1983, p. 3.

22 On the embryo research debate, see Mulkay (1997) and Theodosiou & Johnson (2010).

23 Epidemiologists suggest that the birth prevalence of NTDs in England and Wales has declined significantly since the 1960s (from 3.6 per 1000 births in 1964 to 0.3 per 1000 in 2004), but remain second only to cardiac anomalies as the most common congenital malformations: Morris & Wald (2007).
